# The influence of radio frequency‐based toothbrush on the accumulation of calculus and periodontal health: A randomized double‐blind controlled prospective study

**DOI:** 10.1002/cre2.770

**Published:** 2023-07-29

**Authors:** Juan Khoury, Hadar Z. Giladi, Ofir Ginesin, Eran Gabay, Yaniv Mayer

**Affiliations:** ^1^ Department of Periodontology, School of Graduate Dentistry Rambam Health Care Campus (RHCC) Haifa Israel; ^2^ Faculty of Medicine Technion ‐ Israel Institute of Technology Haifa Israel

**Keywords:** calculus, gingivitis, radio frequency, toothbrush

## Abstract

**Objectives:**

The use of a toothbrush with radio frequency (RF) has shown to be of benefit regarding the reduction of plaque, calculus, and dental staining and improving teeth shade compared to conventional powered and manual toothbrushes. Aim: To evaluate the efficacy of the RF toothbrush in the reduction of calculus accumulation and its effect on periodontal parameters as well as subject satisfaction as compared to an identical sham‐tooth brush.

**Materials and Methods:**

Patients who are under a strict maintenance program were included. Patients were allocated to test (RF toothbrush) or control (sham) randomly and were examined at baseline, one and three months. Clinical photos were taken and a consequential calculus assessment via ImageJ software. Clinical assessment included the following: plaque index (PI), bleeding on probing (BOP), probing pocket depth (PPD), and recession (REC). Patient satisfaction was assessed via a questionnaire.

**Results:**

Fifty‐eight patients (29 control, 29 test) were included. At baseline mean PPD, BOP, PI, REC, and calculus accumulation were similar between the groups. Mean buccal calculus was lower in the test group at one month 4.0% versus 6.7%, *p* < .05. Calculus accumulation within the groups was lower in the test group at 1 and 3 months when compared to baseline at the buccal aspect (2.8% vs. 8.9%, *p* < .05% and 3.8% vs. 8.9%, *p* < .05) and lingual aspect (6.7% vs. 16.5%, *p* < .05% and 8.9% vs. 16.5%, *p* < .05). No statistically significant results were found regarding periodontal parameters PPD, BOP, PI, and REC. No difference was found between groups regarding patient satisfaction.

**Conclusion:**

RF seems to have an additive effect on preventing calculus accumulation on the buccal aspect of anterior mandibular teeth at 1 month. Nevertheless, at 3 months, no difference between the toothbrushes is seen regarding calculus formation and maintaining periodontal health (ClinicalTrials.gov, Identifier NCT04640857).

## 1 INTRODUCTION

Periodontitis is an inflammatory, irreversible disease that affects more than 50% of the population (Petersen & Ogawa, 2012). Gingivitis is a reversible inflammatory situation and is a preliminary stage of periodontal disease (Lang et al., 2009). Although not all gingivitis cases will exacerbate periodontitis, the treatment of gingivitis is a primary prevention strategy as well as a second prevention strategy for the development of periodontal disease (Chapple et al., 2015). The major risk factor of periodontitis is dental plaque accumulation on the tooth surface near the gingiva (Lertpimonchai et al., 2017). Removal and/or plaque control is therefore very important.

Calculus is a layered white or brown colored material. The adherence of calculus to tooth surfaces involves a complex process of chemical bonding that takes advantage of the unique properties of both the tooth surface and calculus. When dental plaque, a biofilm primarily composed of bacteria and extracellular polymeric substances (EPS), is not adequately removed from the tooth surface, it can mineralize into calculus (also known as tartar). The mineralization process involves the integration of calcium and phosphate ions from saliva and gingival crevicular fluid (GCF), which precipitate within the organic matrix of the plaque, transforming it into a hard and tenacious structure (Akcalı & Lang, 2018; Marsh, 2006). The calculus then becomes strongly bound to the tooth surface, primarily through ionic bonds with the hydroxyapatite layer, as well as via interactions with proteins and glycoproteins of the acquired pellicle, a proteinaceous layer formed on the tooth surface. The strong attachment to calculus necessitates professional dental intervention for removal. It often accumulates on the front lower teeth due to food residue and saliva excretion. Serving as a bacterial reservoir, calculus facilitates dental biofilm retention, contributing to inflammation and periodontal disease (Miller et al., 2003; Zader et al., 1960). Furthermore, subgingival calculus is directly correlated with enhanced periodontal attachment loss (White, 1997).

Oral hygiene practice and a strict maintenance program are of major importance in keeping periodontal patients from deteriorating. Adherence to supportive periodontal treatment and compliance have shown a more stable state of the periodontium in general, and specifically in periodontal patients (Costa et al., 2019). Individuals with well‐maintained periodontitis showed a more dysbiotic microbial community than healthy individuals (Lu et al., 2020). Therefore, close monitoring and scheduled maintenance treatment are necessary. The use of an electromagnetic field was shown to be of benefit in promoting the healing of bone fractures (Griffin et al., 2011). In the dental world, it has been studied in several aspects and was found to promote healing around dental implants (Matsumoto et al., 2000; Nayak et al., 2020) as well as fractured long bones in a rabbit model (Nayak et al., 2020). The electromagnetic field lowers osteoclast activity and promotes osteoid formation and angiogenesis (Barnaba et al., 2013; Zhang et al., 2020). Furthermore, it was found that the electromagnetic field has an antimicrobial effect against periopathogens (Faveri et al., 2020). Previous studies have shown that electromagnetic pulses are beneficial in the reduction of plaque and calculus and in the prevention and treatment of gingivitis, in addition to lightning the shade of the teeth and removal of stains (Amaechi et al., 2022; Milleman et al., 2020, 2021; Shehadeh et al., 2021). Radio frequency (RF) energy can indirectly influence covalent and electrostatic forces between molecules through various mechanisms. One indirect way RF energy can affect covalent and electrostatic forces is by generating heat. When RF energy is applied to a material, it can cause the molecules to vibrate and generate thermal energy. This increase in temperature can disrupt weak interactions, such as hydrogen bonds or van der Waals forces, that contribute to the stability of molecular structures (Lai & Singh, 1995). RF‐induced plasma, an ionized gas with free electrons and positive ions, represents another indirect mechanism. RF energy can initiate a plasma state in certain materials, wherein the highly energetic ions and electrons can collide with molecules, breaking bonds and inducing chemical reactions (Stoffels et al., 2008).

Additionally, RF energy can be utilized in certain chemical processes where it indirectly assists in breaking covalent and electrostatic forces. For example, in some types of chemical reactions, RF energy can provide the activation energy needed to initiate the reaction by destabilizing existing bonds and facilitating the formation of new bonds. When RF was compared with a powered toothbrush, the RF test group revealed a statistically significant reduction in calculus (Milleman et al., 2020). When compared to a sonic vibrating toothbrush, RF prevented additional calculus accumulation (Milleman et al., 2021). These results are attributed to the unique RF mechanism.

**Figure 1:**
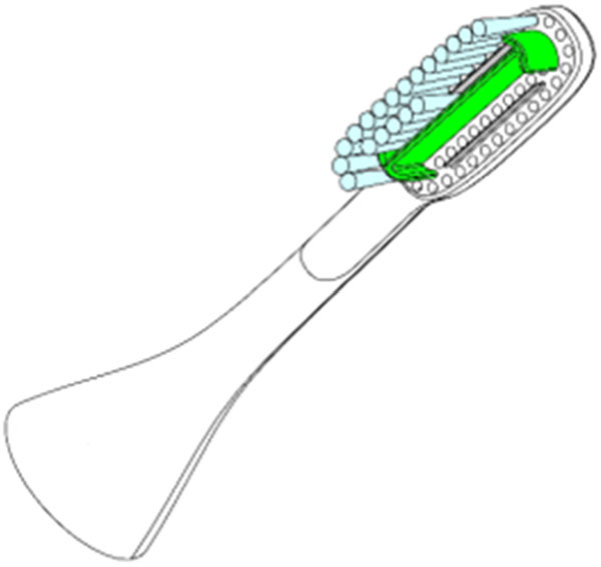
A three‐dimensional drawing of the toothbrush head, exhibiting the silicone strip and the electrodes.

An RF toothbrush, aimed at enhancing dental hygiene and gingival health, uses low‐energy RF power to non‐abrasively cleanse tooth surfaces. It features an oval brush head with side‐to‐side vibration, a silicon strip, and two electrodes that stream energy over the silicon barrier to the teeth while brushing (Figure 1).

RF is an alternating electric current that oscillates at radio frequencies in the range of 3 kHz–300 GHz (Faveri et al., 2020). The technology is meant to bring charged molecules that originate from the toothpaste to the tooth surface, to destabilize electrostatic bonds between the tooth and the impurities, that is, calculus, stains, and plaque attached.

While conventional and electric toothbrushes are able to remove plaque solely, the RF toothbrush technology is unique regarding calculus detaching from dental surfaces. The improved and increased efficacy of the RF toothbrush might be explained due to its technological feature, where the electromagnetic field changes local charges around the tooth, disturbing the electro‐chemical balance of the tooth surface and thus theoretically removing calculus. Therefore, the aim of this study was to investigate the effect of RF toothbrushes on calculus formation. In addition, we checked periodontal inflammatory status as well as patient satisfaction.

Our hypothesis is that the RF toothbrush will lessen calculus accumulation on anterior mandibular teeth between supportive periodontal therapy sessions.

## 2 MATERIALS AND METHODS

### 2.1 Study protocol

The study was approved by Rambam Health Care Campus Ethics Committee (Approval ID: RMB‐0352‐20) and registered at ClinicalTrials.gov (Identifier NCT04640857). The study was conducted in the Department of Periodontology, School of Graduate Dentistry, Rambam Health Care Campus, between December 2020 and August 2022. Patients during supportive periodontal therapy were invited to participate in the study. Patients who fitted to the inclusion criteria and signed an informed consent form were recruited for this prospective, randomized, double‐blind controlled study. Inclusion criteria included patients aged 20–85 years, diagnosed with clinical gingival health with an intact periodontium and stable condition on a reduced periodontium according to the 2017 Classification of Periodontal and Peri‐Implant Diseases and Conditions (Caton et al., 2018), who attend supportive periodontal therapy on a regular basis once every 3 months, and who attended three consecutive maintenance programs; presence of plaque on teeth 32–42 (Plaque index by Silness and Loe 1963 (Löe, 1967)); presence of calculus on buccal and/or lingual on teeth 32–42, and patients that are willing to adhere to research schedules and visits. All patients included in the study were with no periodontal pockets over 4 mm. Exclusion criteria were a systemic or medicinal condition that can affect the healing of the soft hard tissues of the oral cavity; the use of Chlorhexidine mouthwash on a regular basis; patients currently taking systemic antibiotics; chronic use of NSAIDs on a long‐term basis (excluding low dose aspirin); the presence of a pacemaker; pregnant women or planning a pregnancy in the upcoming 12 months; patients with active periodontal disease; one or more crowned anterior mandibular teeth and cigarette smoking > 10 a day.

### 2.2 Study design and allocation

**Figure 2:**
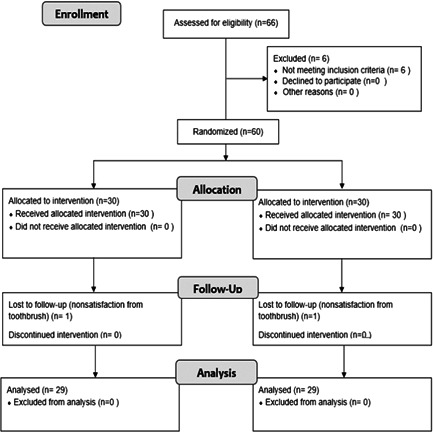
Study flow‐chart.

This is a prospective, double‐blind, randomized, sham‐controlled two‐arm study of a 3‐month duration. Data is reported according to the Consolidated Standards of Reporting (CONSORT) guidelines. One experienced investigator (J.K.) evaluated the subjects and was responsible for the patients' enrollment process following the assessment of the inclusion and exclusion criteria. The study flow chart is reported in Figure 2.

All brushes came with serial numbers (1–60) and were given to the participants by order of recruitment. The toothbrushes were pre‐divided to Group A or B via Research Randomizer as a way to generate random numbers, or in this case, assign the numbers to Groups A or B in a random way. This was done before the study began clinically. Thus, the patients were recruited and given a toothbrush in numerical ascending order, and consequently allocated to Group A or B according to the randomizer. Throughout the study and the analysis of the results, neither the investigators nor the research coordinator knew that Group A was the test and Group B was a sham. The test and sham group were revealed to us after the statistical analysis was performed.

Group A (Test): Radio‐Frequency + Vibration. The patients in this group received a treatment of calculus removal using an ultrasonic device and manual curettes. Oral hygiene instructions were given by the same operator (J.K.) and the use of the toothbrush was demonstrated on a plastic model. The brushing technique instructed was modified bass; a 45° tilt of the bristles into the sulcus, a horizontal scrub of two to three teeth, and finally a sweep in an apico‐coronal direction. The brushing time instructed was a minimum of 2 min, while three vibration strengths were optional. At the end of the meeting, the patients received a vibrating electric toothbrush with active RF technology.

Group B (Control): Vibration. The patients in this group received a treatment of calculus removal using an ultrasonic device and manual curettes. The same oral hygiene instructions were given, and the use of the toothbrush was demonstrated on a plastic model. At the end of the meeting, the patients received a vibrating electric toothbrush without RF technology.

The toothbrushes were identical in shape, size, and vibration. The RF sign was illuminated in both groups to prevent exposure to group allocation but was only activated in the test group.

### 2.3 Outcome variables

An assessment of the soft and hard tissues was done before maintenance treatment at screening (T0), as well as at the follow‐up visits at 4 (T1) weeks and 12 (T2) weeks. All patients have had to exhibit a mild‐moderate inflammatory state of the gingiva (redness/swelling/bleeding on probing) and calculus accumulation around the four anterior lower teeth (31,32,41,42).

**Figure 3:**
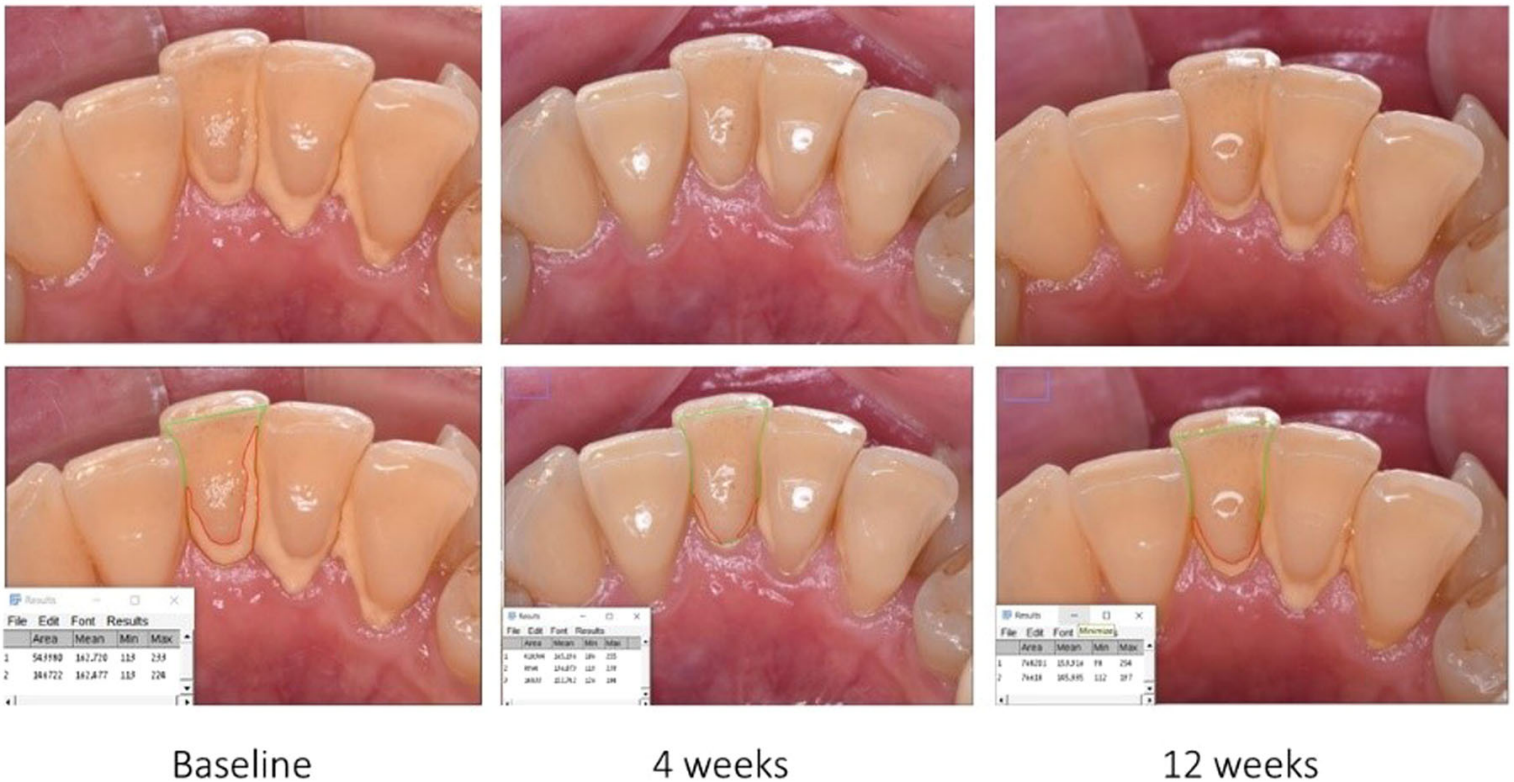
Example of calculus percentage calculation via ImageJ software.

All clinical parameters were evaluated by the same blinded and calibrated examiner (J.K.);

- Clinical photographs—Photographs of buccal and lingual aspects using a professional reflex camera were taken before the clinical measurements and cleaning were done and after 1 month and 3 months. A NIKON D5600 DSLR camera was used, a Nikon AF‐S DX Micro NIKKOR 85 mm f/3,5 G ED VR Lens, and a GODOX ML150 LED Ring flash.
- Plaque Index (PI)—The bacterial plaque index in teeth 31,32,41,42 was recorded at each tooth site on a scale of 0–3, where 0 denotes no plaque, 1 denotes a thin plaque layer at the gingival margin only detectable by scraping with a probe, 2 denotes a moderate layer of plaque along the gingival margin visible to the naked eye in the sulcus, and 3 denotes large amount of plaque along the gingival margin sulcus and interdental spaces.
- Periodontal Pocket Depth (PPD) around teeth—Measured from the mucosal margin to the bottom of the probable pocket using a graduated manual periodontal probe (PCP‐UNC 15; Hu‐Friedy®). Six sites per tooth were evaluated (mesiobucal, midbuccal, distobuccal, mesiolingual, midlingual, and distolingual).
- Bleeding on Probing (BOP)—was recorded dichotomously with either the presence/absence of bleeding within 30 s following probing.
- Gingival recession (REC)—The Cemento–Enamel Junction (CEJ) was the point of reference to the measurements of the buccal and lingual recessions. In cases where there was excessive plaque or calculus accumulation, the measurement was done after its removal to be more accurate.
- Calculus assessment—The measurement of the percentage of the clinical crown (incisal edge to free gingival margin), which is covered with calculus via ImageJ software (National Institutes of Health) as shown in Figure 3.

Assessment of the degree of patient satisfaction: Throughout the research visits, the patients were asked about their level of satisfaction (very high/high/neutral/low/very low) in each of the following topics:

1. The level of satisfaction with the use of the toothbrush.
2. The level of satisfaction regarding the operational aspect of the toothbrush.
3. The level of satisfaction in the improvement in oral hygiene as a result of the use of the toothbrush.

All participants in both groups received toothpaste to use during their participation in the study.

The primary outcome was calculus accumulation. Secondary outcomes were periodontal parameters (PI, PPD, BOP, and REC) and subject satisfaction.

In our study, we rigorously assessed the intra‐observer reproducibility of our measurements to ensure data reliability and precision. Our observer (YM) conducted a series of 10 repeated measurements, the results of which were subjected to a thorough analysis to identify any discrepancies or variations. We utilized both point estimates and a 95% confidence interval to analyze the differences between measurements. Our findings showed high consistency in our methodology, with the intra‐class correlation coefficient (ICC) for calculus percentage measurements achieving remarkably high scores of 0.92 and 0.90. This demonstrates an exceptional level of reproducibility, thereby strengthening the reliability of our measurements. Furthermore, the depth of the periodontal pockets (ppd) was also measured, yielding a mean difference of −0.340 mm with a 95% confidence interval ranging from −0.56 to −0.042 (*p* = .031). These results highlight the high level of precision and repeatability in our methods, emphasizing the trustworthiness of our observations and findings.

### 2.4 Sample size

The number of participants was decided according to a previous paper (Milleman et al., 2020), and the calculation is as follows: *α* 0.05, *β* 0.2, and a delta of 1.95 between groups with a standard deviation of 2.6. According to this data, 28 patients are needed per group (with a confidence level of 95%). The patients were divided into two groups according to the protocol.

A 15% dropout would be possible. If more than five patients from each group will not resume the 3‐month timeline, a supplementary recruitment was done to complete a minimum of 85%. A time window of ±4 months was possible in order for the case to be included in the Treated Per Protocol (TPP) group. A deviation from this time window transferred the patient to the Intended to Treat (ITT) group. The study was approved by Rambam Health Care Campus Ethics Committee (Approval ID: RMB‐0352‐20) and registered at ClinicalTrials.gov (Identifier NCT04640857). Informed consent was obtained from all subjects involved in the study.

### 2.5 Statistical analysis

Data were analyzed using IBM SPSS statistics software version 28.0. (SPSS Inc.). The significance levels were set at .05. Descriptive statistics, including mean, standard deviation, and minimum or maximum ranges, were calculated for all the indices indicated above. A mean, range, and standard deviation were calculated for each value. The comparison between the two groups in each of the specified parameters was done as follows: Continuous measurements were summarized with the count, mean and SD, Median, 25th and 75th percentile minimum and maximum. Categorical variables (gender and smoking) were presented in a contingency table and compared using Fisher exact test. The normality distribution of measures was evaluated using Shapiro and Wilk test, as the majority of measures deviated from the normal distribution nonparametric approach was implemented. Comparison between two groups was evaluated using Mann–Whitney test. A two‐side *p* value less than .05 was considered to define statistical significance. Analyses were carried out using R‐3.6.3 (R Foundation for Statistical Computing).

## 3 RESULTS

Sixty subjects (29 males and 31 females) were recruited for the study. During the study, two patients dropped out (one from Group A and one from Group B), both were females due to loss of follow‐up. The age of the participants ranged from 25 to 80 years, with a mean of 56.5 ± 12.2 years. Five participants were current smokers (<10 cigarettes per day), three in the test group and two in the control (Table 1).

**Table 1:** Baseline demographics and characteristics.

	Radio frequency + Vibration	Vibration	Total	*p*‐Value
Age				.6915
*N*	29	29	58	
Mean (SD)	56.0 (12.8)	57.0 (11.7)	56.5 (12.2)	
Gender				.5999
Female	16 (55.2%)	13 (44.8%)	29 (50.0%)	
Male	13 (44.8%)	16 (55.2%)	29 (50.0%)	
Smoking				.5657
Former smoker	3 (10.3%)	1 (3.4%)	4 (6.9%)	
Nonsmoker	23 (79.3%)	26 (89.7%)	49 (84.5%)	
Smoker	3 (10.3%)	2 (6.9%)	5 (8.6%)	
Calculus buccal				.7439
*N*	29	29	58	
Mean % (SD)	9.8 (3.9)	12.2 (8.3)	11.0 (6.6)	
Calculus lingual				.858
Mean % (SD)	16.3 (7.0)	17.0 (8.0)	16.7 (7.4)	
PPD				.9194
Mean mm (SD)	1.7 (0.2)	1.7 (0.2)	1.7 (0.2)	
BOP				.7202
Mean (SD)	3.4 (0.7)	3.3 (0.9)	3.4 (0.8)	
PI				.494
Mean (SD)	1.9 (0.6)	1.9 (0.7)	1.9 (0.6)	
REC buccal				.5319
Mean mm (SD)	1.5 (1.2)	1.3 (1.2)	1.4 (1.2)	
REC lingual				.1159
Mean mm (SD)	1.6 (1.2)	1.9 (1.4)	1.8 (1.3)	

Abbreviations: BOP, bleeding on probing; *N*, number of participants; PI, plaque index; PPD, periodontal pocket depth; REC, recession; SD, standard deviation.

At baseline, the mean of all teeth was 11 ± 6.6 regarding buccal calculus, 16.7 ± 7.4 regarding lingual calculus, 1.7 ± 0.2 regarding PPD, 3.4 ± 0.8 regarding BOP, 1.9 ± 0.6 regarding PI, 1.4 ± 1.2 regarding buccal REC and 1.8 ± 1.3 regarding lingual REC. No difference was found between the test and control groups at baseline (Table 1).

Clinical parameters postcalculus removal at 4 weeks (T1), reveal a significant difference between groups in the buccal calculus accumulation. The test group has significantly lower values than the control 4.0% ± 2.9 versus 6.7% ± 5.2, respectively (*p* < 0.05) (Table 2). All the rest of the parameters showed no significant difference between groups.

**Table 2:** Characteristics at 4 weeks (T1) postcalculus removal.

4 Weeks (T1)	Radio frequency + Vibration	Vibration	Total	*p*‐Value
Calculus buccal				**.0026**
*N*	29	29	58	
Mean % (SD)	4.0 (2.9)	6.7 (5.2)	5.3 (4.4)	
Calculus lingual				.484
Mean % (SD)	7.4 (5.3)	8.9 (7.5)	8.1 (6.5)	
PPD				.1329
Mean mm (SD)	1.6 (0.3)	1.6 (0.3)	1.6 (0.3)	
BOP				.1116
Mean (SD)	0.3 (0.8)	0.7 (1.0)	0.5 (0.9)	
PI				.5072
Mean (SD)	0.7 (0.6)	0.7 (0.7)	0.7 (0.6)	
REC buccal				.6364
Mean mm (SD)	1.6 (1.2)	1.4 (1.2)	1.5 (1.2)	
REC lingual				.442
Mean mm (SD)	1.7 (1.3)	2.0 (1.5)	1.8 (1.4)	

Abbreviations: BOP, bleeding on probing; N, number of participants; PI, plaque index; PPD, periodontal pocket depth; REC, recession; SD, standard deviation.

Clinical parameters postcalculus removal at 12 weeks (T2) exhibit no significant differences between groups. Nevertheless, lingual calculus accumulation is higher than buccal (12.1% ± 9 vs. 6.8% ± 6.5), respectively (Table 3).

**Table 3:** Characteristics at 12 weeks (T2) postcalculus removal.

12 Weeks (T2)	Radio frequency + Vibration	Vibration	Total	*p*‐Value
Calculus buccal				.2831
*N*	29	29	58	
Mean % (SD)	6.1 (5.5)	7.5 (7.5)	6.8 (6.5)	
Calculus lingual				.5755
Mean% (SD)	11.4 (8.5)	12.8 (9.7)	12.1 (9.0)	
PPD				.166
Mean mm (SD)	1.7 (0.3)	1.7 (0.3)	1.7 (0.3)	
BOP				.924
Mean (SD)	0.7 (1.0)	0.8 (1.2)	0.8 (1.1)	
PI				.5396
Mean (SD)	0.9 (0.7)	0.8 (0.6)	0.9 (0.7)	
REC buccal				.9294
Mean mm (SD)	1.5 (1.3)	1.5 (1.3)	1.5 (1.3)	
REC lingual				.4756
Mean mm (SD)	1.7 (1.4)	1.9 (1.4)	1.8 (1.4)	

Abbreviations: BOP, bleeding on probing; *N*, number of participants; PI, plaque index; PPD, periodontal pocket depth; REC, recession; SD, standard deviation.

A sub‐analysis of the calculus accumulation and changes within each arm is shown in Table 4. The test group reveals a statistical significance regarding calculus accumulation at buccal and lingual aspects between each time point; buccally T0 versus T1 (8.9% vs. 2.8%), T0 versus T2 (8.9% vs. 3.8%) and T1 versus T2 (2.8% vs. 3.8%), respectively (*p* < .05), and lingually T0 versus T1 (16.5% vs. 6.7%), T0 versus T2 (16.5% vs. 8.9%), and T1 versus T2 (6.7% vs. 8.9%), respectively (*p* **<** 0.05) (Table 4).

**Table 4:** Changes in calculus accumulation at T0, T1, and T2 in each group over time.

Calculus accumulation		Radio frequency + Vibration *N* = 29	Vibration *N* = 29
Buccal	T0	8.9% (7.5, 11.5)	8.9% (6.2, 17.5)
	T1	2.8% (2.1, 5.0)	5.3% (4.6, 6.9)
	T2	3.8% (2.7, 8.1)	5.4% (4.2, 8.4)
	T0 vs. T1	**<0.001**	**<0.001**
	T0 vs. T2	**0.001**	**0.001**
	T1 vs. T2	**0.009**	0.400
Lingual	T0	16.5% (10.1, 20.8)	16.8% (11.2, 19.8)
	T1	6.7% (3.3, 9.5)	6.5% (4.5, 10.5)
	T2	8.9% (5.2, 14.4)	9.8% (5.9, 18.2)
	T0 vs. T1	**<0.001**	**<0.001**
	T0 vs. T2	**0.012**	0.147
	T1 vs. T2	**0.002**	**0.004**

*Note*: *N* = number of participants; T0 = baseline; T1 **=** 4 weeks; T2 **=** 12 weeks.

Regarding the control group, at T1, both buccal and lingual aspects demonstrate significantly lower calculus values when compared to baseline; buccally 5.3% versus 8.9% (*p* < .05) and lingually 6.5% versus 16.8% (*p* > .05). At T2, the buccal aspect reveals less buccal accumulation when compared with baseline 5.4% versus 8.9% (*p* < .05), yet there is no improvement in the calculus accumulation between T1 and T2, 5.3% versus 5.4%, respectively (*p* = 0.4). The lingual aspect of the control group shows no improvement in calculus accumulation when comparing T0 with T2, 16.8% versus 9.8%, respectively (*p* = 0.147). Yet, a statistical difference is evident between T1 and T2, 6.5% versus 9.8%, respectively (*p* < .05) (Table 4). Both groups exhibit higher calculus values at T2 when compared with T1.

Regarding patients' satisfaction, there was no difference between groups in any of the questions. The satisfaction of most of the participants was high or very high in both groups (Table 5).

**Table 5:** Satisfaction questionnaire table.

		RF+vib (*N* = 29)	Vib (*N* = 29)	Total (*N* = 58)	*p*‐Value
Week 4					.5142
Q1	Very high	8 (25.8%)	3 (9.7%)	11 (17.7%)	
	High	10 (32.3%)	14 (45.2%)	24 (38.7%)	
	Neutral	9 (29.0%)	11 (35.5%)	20 (32.3%)	
	Low	2 (6.5%)	1 (3.2%)	3 (4.8%)	
	Very low	2 (6.5%)	2 (6.5%)	4 (6.5%)	
					.7561
Q2	Very high	5 (16.1%)	6 (19.4%)	11 (17.7%)	
	High	9 (29.0%)	13 (41.9%)	22 (35.5%)	
	Neutral	11 (35.5%)	7 (22.6%)	18 (29.0%)	
	Low	4 (12.9%)	3 (9.7%)	7 (11.3%)	
	Very low	2 (6.5%)	2 (6.5%)	4 (6.5%)	
					.7701
Q3	Very high	4 (12.9%)	5 (16.1%)	9 (14.5%)	
	High	12 (38.7%)	10 (32.3%)	22 (35.5%)	
	Neutral	9 (29.0%)	13 (41.9%)	22 (35.5%)	
	Low	4 (12.9%)	2 (6.5%)	6 (9.7%)	
	Very low	2 (6.5%)	1 (3.2%)	3 (4.8%)	
					.0415
Q4	Electric brush is better	26 (83.9%)	17 (54.8%)	43 (69.4%)	
	Manual brush is better	3 (9.7%)	8 (25.8%)	11 (17.7%)	
	No difference	2 (6.5%)	6 (19.4%)	8 (12.9%)	
Week 12					.963
Q1	Very high	10 (33.3%)	9 (31.0%)	19 (32.2%)	
	High	9 (30.0%)	11 (37.9%)	20 (33.9%)	
	Neutral	7 (23.3%)	7 (24.1%)	14 (23.7%)	
	Low	3 (10.0%)	2 (6.9%)	5 (8.5%)	
	Very low	1 (3.3%)	0 (0.0%)	1 (1.7%)	
					.4548
Q2	Very high	9 (30.0%)	10 (34.5%)	19 (32.2%)	
	High	9 (30.0%)	11 (37.9%)	20 (33.9%)	
	Neutral	9 (30.0%)	4 (13.8%)	13 (22.0%)	
	Low	2 (6.7%)	4 (13.8%)	6 (10.2%)	
	Very low	1 (3.3%)	0 (0.0%)	1 (1.7%)	
Q3	Very high	6 (20.0%)	5 (17.2%)	11 (18.6%)	
	High	11 (36.7%)	12 (41.4%)	23 (39.0%)	
	Neutral	10 (33.3%)	10 (34.5%)	20 (33.9%)	
	Low	3 (10.0%)	2 (6.9%)	5 (8.5%)	
	Very low	0 (0.0%)	0 (0.0%)	0 (0.0%)	
					.2084
Q4	Electric brush is better	26 (86.7%)	19 (65.5%)	45 (76.3%)	
	Manual brush is better	2 (6.7%)	5 (17.2%)	7 (11.9%)	
	No difference	2 (6.7%)	5 (17.2%)	7 (11.9%)	

## 4 DISCUSSION

Plaque accumulates on non‐shedding tissues in the oral cavity, and within 2 weeks of its formation, minerals from the saliva and crevicular fluid precipitate in the dental plaque and form calculus (Akcalı & Lang, 2018). The rough and hardened surface of the calculus provides an ideal ground for further plaque formation and build‐up (Aghanashini et al., 2016). Manual or power toothbrushing is recommended as a primary means of reducing plaque and gingivitis (Sanz et al., 2015). The use of a powered toothbrush may be considered as an alternative to manual toothbrushing for periodontal maintenance patients, taking into account patients' needs and preferences (Sanz et al., 2015). Nevertheless, this hardened material is attached to the tooth surface and cannot be removed with a conventional (mechanical) or powered toothbrush. In our study, the RF‐activated toothbrush showed significant calculus reduction at 1 month when compared with the control group where the RF was inactivated; also an improvement in bleeding on probing was shown. Nevertheless, during the 3‐month period, no difference was shown between the groups regarding calculus formation and maintaining periodontal health.

In general, the periodontal parameters BOP, PI, and PD all went down in the first month of the study. One might explain the results due to the patient's awareness of taking part in the study, known as the Hawthorne effect, (Adair et al., 1989) thus being under strict oral hygiene habits. The same toothbrush with similar vibration power levels was given to the control and test group. The only difference was the activation of the radio frequency, which both the patient and us, the researchers, were blinded to. The results reveal having an end result of significantly less calculus at buccal and lingual aspects of the anterior teeth after 1 month in both groups when compared with the baseline value. Nevertheless, a significant difference in calculus accumulation between the groups after 1 month of participating in the study was evident as well, showing an added benefit to the use of an RF toothbrush (test group). Comparison of the same toothbrush with or without RF verifies the efficacy of the RF, clinically resulting in significantly less accumulation of calculus in the buccal aspect in the test group at 1 month. Nevertheless, the fact that no statistical difference was seen between groups at 3 months, might hint that the more calculus accumulates, the effect of the RF lessens.

While imageJ software is used in many assessments of laboratory methods, according to our knowledge, this is the first time using a digital method to measure the amount of calculus accumulation. Although several manual calculations of calculus accumulation are optional; the Volpe‐Manhold calculus index, (Barnett et al., 1989) OHI‐S index calculus score, (Greene & Vermillion, 1964) calculus surface index (CSI), (Dhingra & Vandana, 2011; Ennever et al., 1961) calculus surface severity index (CSSI), (Dhingra & Vandana, 2011) calculus component of periodontal disease, (Ramfjord, 1967) and the marginal line calculus index, (Mühlemann & Villa, 1967) and the results are based on the subjective clinical estimation of the practitioner. The use of ImageJ technology (Schindelin et al., 2015) to assess the percentage of calculus on the teeth is a more objective and precise way.

The results of this study provide evidence for the unique technological feature of the use of electromagnetic fields that utilize RF energy that streams onto the tooth surface during brushing. Since the introduction of power toothbrushes in the early 1960s, they have undergone many technological advances in design and bristle motion, including rotation, oscillation, and sonic vibration. Powered toothbrushes can be categorized as mechanical, sonic, or ionic (Penick, 2004). While a powered mechanical toothbrush works with rotating or oscillating heads, the sonic one emits sound waves as an addition to the movement, and the ionic one works on negative and positive electrical charges (Penick, 2004). Regarding powered toothbrushes, clinical studies indicate a benefit on plaque formation and gingival inflammation (Deacon et al., 2010; Elkerbout et al., 2020; Thomassen et al., 2022). No beneficial effect on calculus was reported in any of the studies. In addition, most of the clinical evidence concludes that the performance of a powered toothbrush does not differ from a manual one regarding calculus accumulation (Deery et al., 2004). RF toothbrush does not provide actual movement of the bristles, but rather in a side‐to‐side vibration mode similar to the streaks of a manual toothbrush and as opposed to the oscillating rotating head of the conventional powered toothbrush. The vibration can be activated or not according to the user's preference. Therefore, a brushing technique should be used; we recommend the modified bass method as it was shown to be superior to other toothbrushing practices in supragingival plaque removal (Janakiram et al., 2020; Poyato‐Ferrera et al., 2003).

The improved and increased efficacy of the RF toothbrush might be explained due to its technological feature, where the electromagnetic field changes local charges around the tooth, disturbing the electro‐chemical balance of the tooth surface and thus theoretically removing calculus. Utilizing RF energy, an alternating electrical current that streams back and forth between two electrodes, providing a localized effect that is limited to the surface of the teeth. The high frequency of the alternating current allows it to safely increase the electrical power, hence achieving more significant results. Furthermore, the RF alternating current streams close to the tooth, bringing the charged molecules that are present in the toothpaste close to the tooth surface, eventually changing the chemical environment around it.

It is not quite clear if the participants in Milleman et al. (2020) had their calculus removal before the study or after baseline measurements. Nevertheless, the RF toothbrush showed positive results of less calculus accumulation when compared to the electric toothbrush. In addition, the study of the RF toothbrush by Milleman and colleagues from 2021, reveals significant calculus reductions compared to a powered toothbrush, while maintaining initial calculus levels and preventing additional calculus accumulation. The effect of RF on calculus accumulation is similar to the results that we presented in our study.

While we did not assess the effect of RF toothbrushes on teeth whitening and removal of stains, three studies, Milleman et al. (2020), Shehadeh et al. (2021), and Amaechi et al. (2022), have all demonstrated significant benefits in the reduction of teeth stains and whitening of teeth shade. Milleman et al. (2020) and Amaechi et al. (2022) compared the RF toothbrush to a conventional electric toothbrush, while Shehadeh et al. (2021) evaluated the effect of an RF toothbrush without mechanical vibration.

Crowded anterior teeth were included in the assessment and were not an exclusion criterion. It should be taken into consideration that they are harder to maintain and thus accumulate calculus more easily.

The single clinical significance was of the buccal aspect at 1 month. Nevertheless. both groups showed an improvement in calculus accumulation. This can be attributed to the novelty effect that may be influencing these early results.

Additional research is necessary to affirm the effectiveness of the RF toothbrush and its comparative advantage in preventing calculus formation and accumulation of overpowered or manual toothbrushes. While our study juxtaposed the use of the same toothbrush with and without RF, subsequent studies should incorporate two further comparative groups: manual and powered toothbrushes.

## 5 CONCLUSIONS

While RF appears to contribute to the prevention of calculus accumulation on the buccal aspects of the lower teeth after 1 month, the novelty effect may be influencing these early results. Over a longer duration, there seems to be no discernable difference between RF and regular toothbrushes concerning calculus formation and periodontal health maintenance.

## AUTHOR CONTRIBUTIONS

*Conceptualization*: Yaniv Mayer. *Methodology*: Yaniv Mayer and Hadar Zigdon Giladi. *Software*: Juan Khoury and Ofir Ginesin. *Validation*: Yaniv Mayer, Juan Khoury, Eran Gabay, and Hadar Z. Giladi. *Formal analysis*: Juan Khoury and Ofir Ginesin. *Investigation*: Juan Khoury and Yaniv Mayer. *Resources*: Yaniv Mayer. *Data curation*: Yaniv Mayer, Juan Khoury, and Eran Gabay. *Writing original draft preparation*: Juan Khoury and Yaniv Mayer. *Writing review and editing*: Juan Khoury, Yaniv Mayer, and Hadar Zigdon Giladi. *Visualization*: Juan Khoury and Ofir Ginesin. *Supervision*: Hadar Z. Giladi and Yaniv Mayer. *Project administration*: Yaniv Mayer. *Funding acquisition*: Yaniv Mayer. All authors have read and agreed to the published version of the manuscript.

## CONFLICT OF INTEREST STATEMENT

The authors declare no conflict of interest.

## Data Availability

Data is available on request due to privacy/ethical restrictions. Data is contained within the article.
